# Prophylactic Administration of a Bacteriophage Cocktail Is Safe and Effective in Reducing Salmonella enterica Serovar Typhimurium Burden *in Vivo*

**DOI:** 10.1128/spectrum.00497-21

**Published:** 2021-08-25

**Authors:** Quentin Lamy-Besnier, Lorenzo Chaffringeon, Marta Lourenço, Rayford B. Payne, Jimmy T. Trinh, Jennifer A. Schwartz, Alexander Sulakvelidze, Laurent Debarbieux

**Affiliations:** a Department of Microbiology, Institut Pasteur, Paris, France; b Université de Paris, Paris, France; c Sorbonne Université, INSERM, Centre de Recherche St Antoine, UMRS_938, Paris, France; d Sorbonne Université, Collège Doctoral, Paris, France; e Intralytix, Inc., Columbia, Maryland, USA; University of Pittsburgh School of Medicine

**Keywords:** foodborne disease, gnotobiotic model, epidemic, prevention, food-borne disease

## Abstract

Nontyphoidal *Salmonella* bacteria are the causative agent of salmonellosis, which accounts for the majority of foodborne illness of bacterial etiology in humans. Here, we demonstrate the safety and efficacy of the prophylactic administration of a bacteriophage preparation termed FOP (foodborne outbreak pill), which contains lytic phages targeting *Salmonella* (SalmoFresh phage cocktail), Shiga toxin-producing Escherichia coli (STEC), and Listeria monocytogenes, for lowering *Salmonella* burdens in OMM^12^ gnotobiotic mice. Prophylactic administration of FOP significantly reduced the levels of *Salmonella* in feces and in intestinal sections compared to the levels in controls. Moreover, the overall symptoms of the disease were also considerably lessened. Dose-dependent administration of FOP showed that phage amplification reached similarly high levels in less than 48 h independent of dose. In addition, 16S rRNA gene analysis showed that FOP did not alter the intestinal microbiota of healthy OMM^12^ mice and reduced microbiota perturbations induced by *Salmonella*. FOP maintained its full potency against *Salmonella* in comparison to that of SalmoFresh, its *Salmonella*-targeting component phages alone. Altogether, the data support that preventive administration of FOP may offer a safe and effective approach for reducing the risk of foodborne infections caused by *Salmonella* and, potentially, other foodborne bacteria (namely, STEC and L. monocytogenes) targeted by the FOP preparation.

**IMPORTANCE** Foodborne bacterial infections cause worldwide economic loss. During an epidemic, the use of antibiotics to slow down the spread of the disease is not recommended because of their side effects on the resident microbiota and the selection of antibiotic-resistant bacteria. Here, we investigated the potential for the prophylactic administration of bacteriophages (viruses infecting bacteria) to reduce the burden of *Salmonella in vivo* using mice colonized by a synthetic microbiota. We found that the repeated administration of bacteriophages was safe and efficient in lowering the *Salmonella* burden. Perturbations of the microbiota by the *Salmonella* infection were also reduced when mice received bacteriophages. Altogether, these data support the use of bacteriophages as a prophylactic intervention to lower the spread of foodborne epidemics.

## INTRODUCTION

Foodborne illnesses are a major cause of morbidity and mortality, with an estimated 600 million foodborne infections and >400,000 deaths worldwide in 2010 (1, 2). The majority of foodborne disease (FBD) is caused by bacterial pathogens, where nontyphoidal *Salmonella* (NTS) strains are among those most commonly associated with invasive disease (3) and gastroenteritis (4). NTS infections are consistently the leading cause of an estimated 80 million cases of FBD worldwide, and approximately 1 million cases of FBD occur in the United States each year. NTS strains are Gram-negative, facultative, anaerobic bacteria consisting of multiple serovars of Salmonella enterica subsp. *enterica*. *Salmonella* infections (i.e., salmonellosis) in humans typically are acquired during transmission from the consumption of contaminated food or water, often from animal sources (5, 6).

Salmonellosis, a form of gastroenteritis, is often self-limiting, resulting in nausea, abdominal cramps, vomiting, and diarrhea that, while often lasting several days in healthy individuals, can lead to serious and deadly complications in vulnerable populations, such as the immunocompromised, elderly, or very young (7, 8). Salmonellosis may be treated using broad-spectrum antibiotics, but recent developments, including antibiotic resistance, have exposed limitations and risks with this approach (9–13). Thus, considerable effort is increasingly directed at finding prevention strategies to lower the dissemination of foodborne pathogens in a more specific manner (14).

Bacteriophages (or phages) offer one such approach. Phages are bacterial viruses that, unlike antibiotics, are highly specific and effective at killing their targeted host bacteria. In addition, the mechanisms by which antibiotics and phages kill bacteria and, conversely, the mechanisms of bacterial resistance to antibiotics or phages are fundamentally different (15–17). There is a long history of using phages to treat bacterial infections in humans, namely, phage therapy (18, 19), and currently, the number of successful compassionate treatments is increasing worldwide (20, 21). In contrast, the prophylactic use of phages remains underevaluated, in particular in the field of FBD.

Here, we utilize gnotobiotic mice (OMM^12^) to examine the tolerability and efficacy of the prophylactic administration of a phage preparation to target an intestinal foodborne pathogen. The OMM^12^ mice harbor a defined and stable intestinal community composed of 12 bacterial strains of mouse origin, which represent the five most prevalent and abundant phyla of the native laboratory mouse intestine (22). Importantly, when challenged by the human foodborne pathogen Salmonella enterica subsp. *enterica* serovar Typhimurium, OMM^12^ mice exhibited symptoms similar to those of salmonellosis in humans, without the need to use an antibiotic treatment to favor intestinal colonization, making these mice a relevant model for assessing the prophylactic impact of phages (22, 23).

We evaluated two phage preparations, SalmoFresh and FOP (foodborne outbreak pill), that target *Salmonella* Typhimurium. SalmoFresh is a cocktail of 6 phages targeting multiple *Salmonella* strains, and FOP is a cocktail of 15 phages that includes those from SalmoFresh, 6 phages targeting Listeria monocytogenes strains, and 3 phages targeting Shiga toxin-producing Escherichia coli (STEC) strains, including E. coli O157:H7 (24). FOP was equally as effective as SalmoFresh in reducing the *Salmonella* burden in feces and intestinal sections and in reducing symptoms of the disease. The prophylactic administration of FOP to healthy mice did not perturb the native gut microbiome, and FOP alone did not induce a gut inflammatory response as measured by the biomarker lipocalin-2. Altogether, these observations show that the prophylactic administration of FOP is both safe and effective to reduce *Salmonella* burdens *in vivo*.

## RESULTS

### SalmoFresh delays the burden of *Salmonella* in OMM^12^ mice.

**FIG 1:**
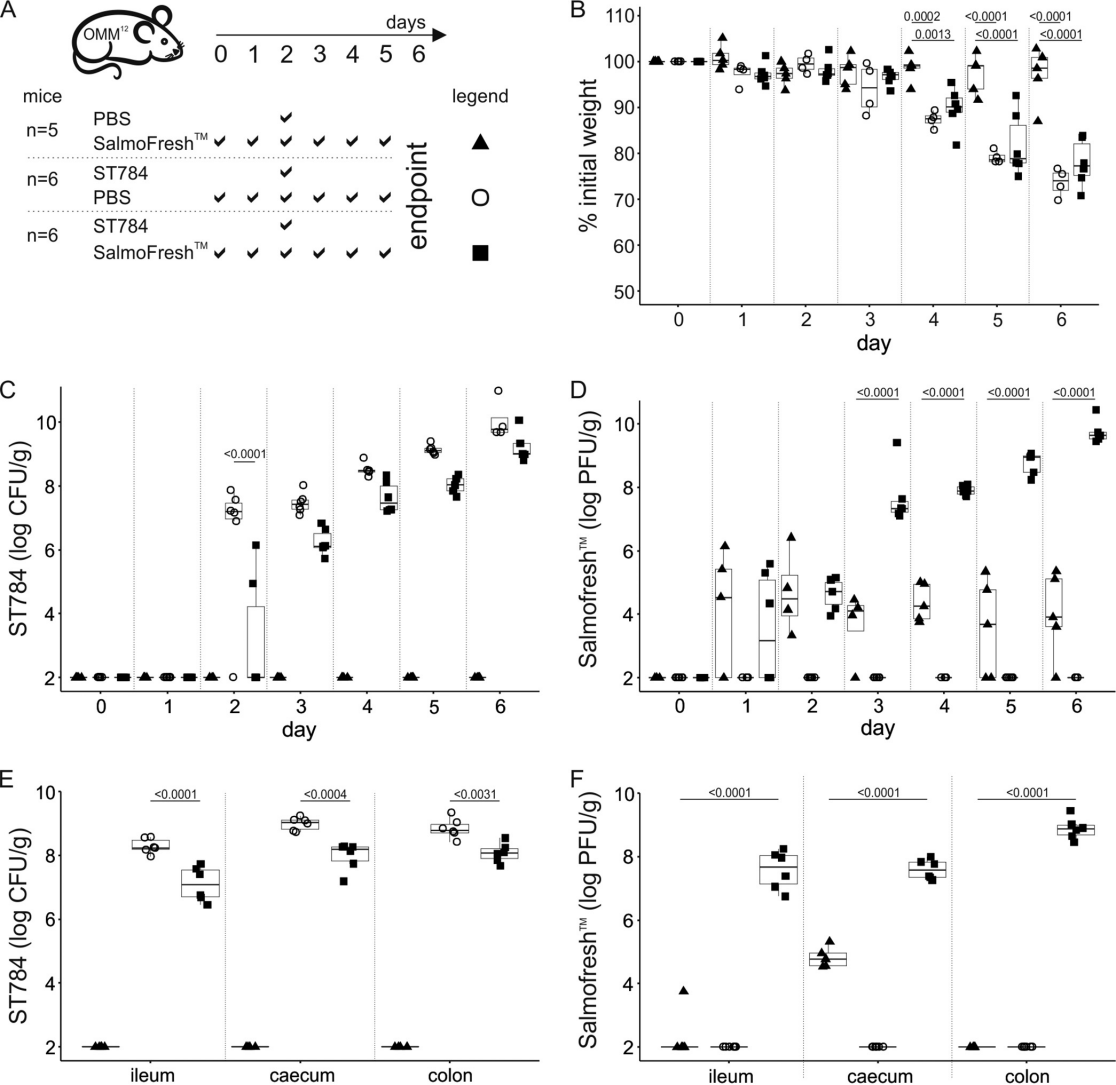
SalmoFresh delays the burden of *Salmonella* after challenge in OMM^12^ mice. (A) Experimental design. Groups of 4 to 6 OMM^12^ mice were orally administered PBS or SalmoFresh (1 × 10^9^ PFU) on the indicated days. On day 2, mice were challenged with ST784 (1 × 10^8^ CFU) or a PBS control as indicated. (B) Mice were weighed daily. Shown are the percentages of weight loss compared to starting weights of OMM^12^ mice over time. (C and D) The amounts of ST784 (CFU) (C) and phages (PFU) (D) in OMM^12^ mouse feces were quantitated daily (day 2 corresponds to 3 h after ST784 challenge). (E and F) Intestinal organs were collected on day 6 and homogenized, and the amounts of ST784 (CFU) (E) and phage (PFU) (F) were quantitated. Statistical analyses are described in Materials and Methods and reported in Table S1.

To assess the impact of the prophylactic administration of phage products on the burden of *Salmonella in vivo*, we first evaluated SalmoFresh, a product that contains six lytic phages (Fig. 1A and Table 1) (24, 25). Animals (3 groups) received for 2 days (days 0 and 1) either phosphate-buffered saline (PBS) (1 group) or SalmoFresh (2 groups) administered twice a day by oral gavage to reduce variability between animals and to ensure appropriate dosing and efficient delivery of the treatments and control. On day 2, the PBS group and one of the SalmoFresh groups received a single administration of *Salmonella* Typhimurium strain ST784 (1 × 10^8^ CFU by oral gavage) (referred to as the ST784 PBS and ST784 SalmoFresh groups), while the other SalmoFresh group received PBS as a SalmoFresh control (referred to as the PBS SalmoFresh group). SalmoFresh (1 × 10^9^ PFU/dose, quantified on strain ST784 prior to use) and PBS were then administered to the respective groups by oral gavage twice a day for an additional 4 days (from day 2 to day 5). Of note, four of the six phages included in SalmoFresh infect strain ST784 with similar efficiencies *in vitro* (see Materials and Methods). The single oral administration of ST784 in OMM^12^ mice receiving PBS (ST784 PBS group) led to a significant loss of weight compared to the weight loss in uninfected mice (PBS SalmoFresh group), starting 48 h post-ST784 administration and lasting two more days before animals reached the experimental endpoint (loss of 25% of their initial weight) (Fig. 1B and Table S1). Infected animals that received SalmoFresh twice a day (ST784 SalmoFresh group) displayed significant weight loss compared to the weight loss in uninfected mice (PBS SalmoFresh group). No significant weight variations were observed between ST784 PBS and ST784 SalmoFresh groups post-ST784 administration (Fig. 1B and Table S1). Additionally, symptoms of the disease (fur appearance, mobility, weight loss, and fecal consistency) were delayed in ST784 SalmoFresh animals compared to the onset in the ST784 PBS controls (Table 1). Concomitant to these observations, the levels of *Salmonella* in fecal samples increased steadily over time in both infected groups (Fig. 1C and Table S1). However, this increase was delayed by approximately 24 h in the ST784 SalmoFresh group and trended lower than in the ST784 PBS group (Fig. 1C and Table S1). This suggests that phages were able to infect ST784 *in vivo*, which was confirmed by the highly significant difference in the fecal phage levels when comparing uninfected and infected groups receiving SalmoFresh, which culminated with a 4-log difference by day 6 (Fig. 1D). Intestinal gut sections (ileum, cecum, and colon) were collected at day 6, and the *Salmonella* and phage levels were measured (Fig. 1E and F). Significant differences in *Salmonella* counts between the ST784 SalmoFresh and ST784 PBS groups were confirmed in all gut sections (Fig. 1E and Table S1). Phage levels in all three gut sections from ST784 SalmoFresh mice reached 10^8^ PFU/g of feces, while in PBS SalmoFresh mice, the levels only reached 10^4^ PFU/g in the cecum and were below the threshold of detection in both the ileum and colon (Fig. 1F). Phages were undetectable in the gut sections of the ST784 PBS group. These data demonstrate that the prophylactic administration of phages delays the burden of *Salmonella* in OMM^12^ mice. They are congruent with results previously obtained when testing SalmoFresh to reduce *Salmonella* contamination on fresh products (25). Additionally, the repeated administration of SalmoFresh in uninfected mice (PBS SalmoFresh group) did not impact their overall health (i.e., behavior and weight), showing that in the absence of their bacterial target, phages are innocuous.

**TABLE 1:** SalmoFresh reduces disease symptoms associated with the burden of *Salmonella* in OMM^12^ mice

Treatment group^*a*^	No. of mice that were^*b*^:		Treatment		Challenge		Clinical signs and symptoms on day^*d*^:						
	M	F	SF^*c*^	PBS	ST784	PBS	0	1	2	3	4	5	6
ST784 SalmoFresh	2	−	+	−	+	−	None	None	None	None	None	*Mild*	Mod
	−	4	+	−	+	−	None	None	None	None	None	None	Mod
PBS SalmoFresh	2	−	+	−	−	+	None	None	None	None	None	None	None
	−	3	+	−	−	+	None	None	None	None	None	None	None
ST784 PBS	2	−	−	+	+	−	None	None	None	None	None	Mod	Mod
	−	4	−	+	+	−	None	None	None	None	Mod	Mod	**Sev**

a Treatment groups were as shown in Fig. 1A.

b M, male; F, female.

c SF, SalmoFresh.

d Clinical signs and symptoms over time (days 0 to 6) are scored based on animal behavior (e.g., activity, hunching) and consistency of fecal pellets (e.g., formed/no liquid, formed/liquid, liquid) as follows: None, no signs of disease; *Mild*, 50% of animals in group exhibited mild signs of disease; Mod, all animals in group exhibited moderate disease; **Sev**, all animals in group exhibited severe disease.

### FOP strongly reduces *Salmonella* burden *in vivo*.

**FIG 2:**
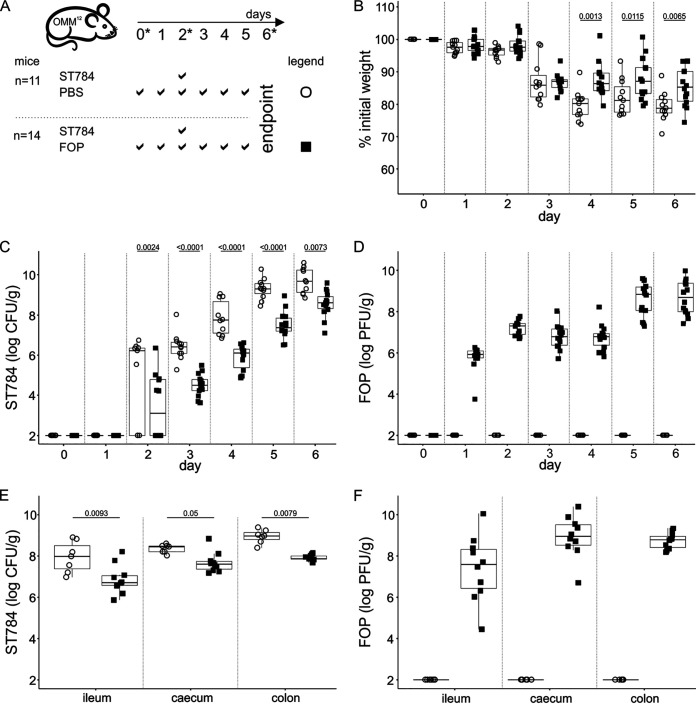
FOP reduces *Salmonella* burden *in vivo*. (A) Experimental design. OMM^12^ mice were orally administered PBS or FOP (1 × 10^9^ PFU) on the indicated days. On day 2, all mice were challenged with strain ST784 (1 × 10^8^ CFU). Stars indicate the time points at which samples were taken for the 16S rRNA gene analysis reported in Fig. 3. (B) Mice were weighed daily. Shown are the percentages of weight loss compared to starting weights of OMM^12^ mice over time. (C and D) The amounts of ST784 (CFU) (C) and phages (PFU) (D) in OMM^12^ mouse feces were quantitated daily (day 2 corresponds to 3 h after ST784 challenge). (E and F) Intestinal organs were collected on day 6, and the amounts of ST784 (CFU) (E) and phages (PFU) quantitated. Statistical analyses are described in Materials and Methods and reported in Table S2.

Using an experiment similar to the one described above, we next assessed the impact of FOP, which contains a combination of products including SalmoFresh (Fig. 2A and Table 2). In two independent experiments, two groups of mice were administered by oral gavage either FOP (1 × 10^9^ PFU/dose; *n* = 14) or PBS (*n* = 11) twice a day for 6 days. Both groups were challenged with ST784 (1 × 10^8^ CFU) on day 2 (ST784 PBS and ST784 FOP groups). We observed in both groups a median weight loss of approximately 10% of their initial weights within 24 h after *Salmonella* administration (Fig. 2B and Table S2). During the following 3 days (days 3 to 5), the median weight loss increased to 20% in the ST784 PBS group, while it stayed roughly stable in the ST784 FOP group (Fig. 2B and Table S2). Fecal pellets collected 3 h after *Salmonella* administration showed that in the ST784 PBS group, *Salmonella* levels reached 10^6^ CFU/g of feces in all but two mice (Fig. 2C and Table S2). This was in sharp contrast to the ST784 FOP group, in which only 6 of 14 mice had *Salmonella* levels above the detection threshold (Fig. 2C and Table S2). During the next 4 days (days 3 to 6), the initial impact of the FOP product was significantly maintained, despite a progressive increase of fecal *Salmonella* levels, where the *Salmonella* burden in the ST784 FOP group was >1-log lower than in the ST784 PBS group (Fig. 2C and Table S2). Additionally, clinical signs and symptoms were significantly reduced and delayed in the ST784 FOP group compared to the clinical signs and symptoms in the ST784 PBS group (Table 2). Fecal phage levels were approximately 10^7^ PFU/g 3 h after *Salmonella* administration, remained stable during the next 2 days (days 3 and 4), and increased up to 10^8^ to 10^10^ PFU/g during the last 2 days (days 5 and 6), which is a pattern expected from continuous infection of increasing levels of *Salmonella* in the intestine (Fig. 2D). The levels of both *Salmonella* and phages in gut sections examined at day 6 were in agreement with the observations presented in Fig. 2C and D, where smaller amounts of *Salmonella* were measured in the ST784 FOP group than in the ST784 PBS group (Fig. 2E and Table S2), together with concomitant elevated levels of phages (Fig. 2F).

**TABLE 2:** FOP reduces disease symptoms associated with the burden of *Salmonella* in OMM^12^ mice

Treatment group^*a*^	No. of mice that were^*b*^:		Treatment		Clinical signs and symptoms on day^*c*^:						
	M	F	FOP	PBS	0	1	2	3	4	5	6
ST784 FOP	6	−	+	−	None	None	None	None	None	None	*Mild*
	−	8	+	−	None	None	None	None	None	None	*Mild*
ST784 PBS	6	−	−	+	None	None	None	None	None	Mod	**Sev**
	−	5	−	+	None	None	None	None	*Mild*	Mod	Mod

a Treatment groups were as shown in Fig. 2.

b M, male; F, female.

c Clinical signs and symptoms over time (days 0 to 6) are scored based on animal behavior (e.g., activity, hunching) and consistency of fecal pellets (e.g., formed/no liquid, formed/liquid, liquid) as follows: None, indicates no signs of disease; *Mild*, indicates 50% of animals in group exhibited mild signs of disease; Mod, indicates all animals in group exhibited moderate disease; **Sev**, indicates 50% of animals in group exhibited severe disease.

An independent experiment was performed with uninfected OMM^12^ mice to assess the safety of repeated administration of FOP, evaluated by observation of mouse behavior and measures of weight and fecal levels of lipocalin-2, a marker of intestinal inflammation (26), over time (Fig. S1A). When comparing groups of mice that received either FOP or PBS, we observed that all animals lost a slight amount of weight in both groups, which was attributed to repeated handling (i.e., twice-daily gavage) (Fig. S1B). No changes in overall health were observed (i.e., fur appearance, mobility, and fecal consistency). Fecal levels of phages were high and remained stable over time (Fig. S1C). Fecal levels of lipocalin-2 were quantitated before and 24 h and 96 h after FOP or PBS administration (Fig. S1D). Overall, lipocalin-2 levels remained below the threshold of inflammation (5 ng/g of feces [26]), and no increases were observed in the FOP group compared to the PBS group, indicating that FOP does not induce gastrointestinal tract inflammation (Fig. S1D).

### The FOP treatment lowers *Salmonella*-induced perturbation of the bacterial consortium of the OMM^12^ mice.

**FIG 3:**
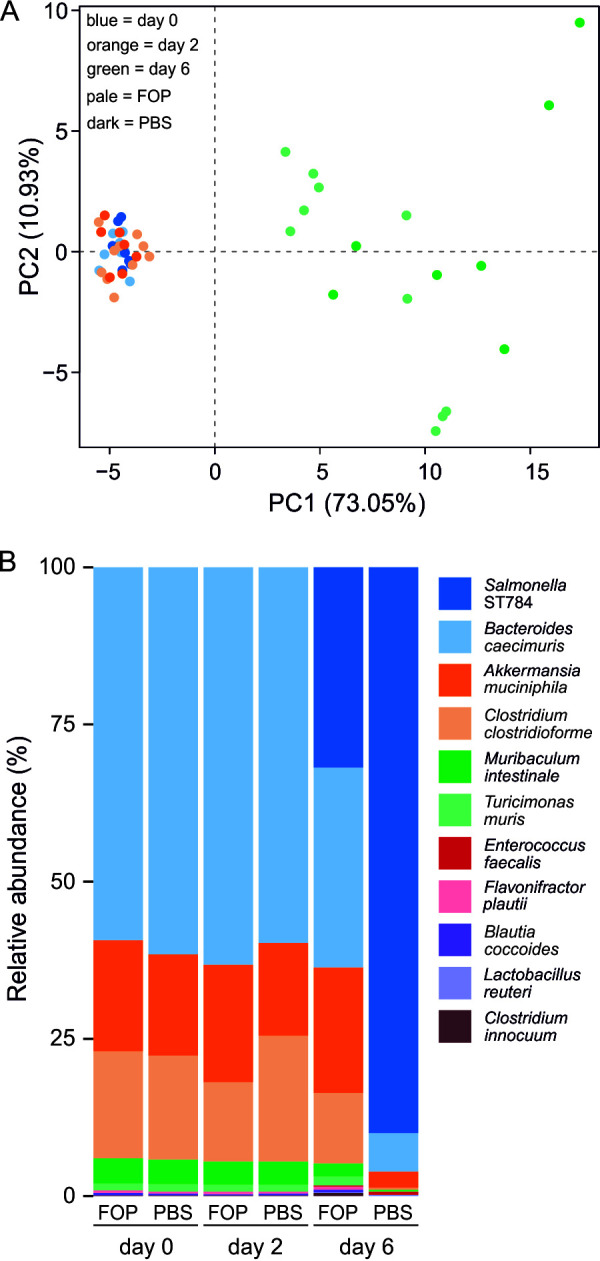
*Salmonella* infection disturbs the microbiota in OMM^12^ mice but FOP does not. (A) Shown is a principal-component analysis (PCA) plot of 16S rRNA gene data obtained from fecal pellets taken at days 0 (baseline), 2 (just before the ST784 challenge), and 6 (before sacrifice) from OMM^12^ mice that received FOP (*n* = 10) or PBS (*n* = 7). All animals were infected with strain ST784 on day 2 (see Fig. 2A for the experimental design). (B) The relative abundances of the ST784 challenge strain and OMM^12^ mouse origin bacteria from the fecal samples are shown. A comparison of log_2_-fold changes of 16S rRNA gene read abundances between days and conditions with statistical values is given in Table S3. Note that only 10 of the 12 mouse origin bacteria are detectable by either 16S rRNA gene or quantitative PCR (qPCR), as reported previously (31).

We next assessed whether the prophylactic administration of the FOP product to *Salmonella*-infected OMM^12^ mice would affect the relative abundances of the resident bacteria of the gut. Fecal DNA was extracted from samples (Fig. 2C) at three time points: before FOP or PBS administrations (day 0), before ST784 challenge (day 2), and before sacrifice on day 6. Samples were subjected to 16S rRNA gene amplification and sequencing (Fig. 3A). No significant differences in bacterial abundance were seen between day 0 and day 2 for each of the PBS and FOP groups of animals. Additionally, no significant differences in bacterial abundance were seen between the FOP or PBS groups on day 2 prior to the bacterial challenge (Fig. 3B and Table S3). As expected, *Salmonella* bacteria were substantially more abundant in day 6 samples but significantly lower in ST784 FOP than in ST784 PBS samples, further demonstrating the efficacy of FOP in reducing *Salmonella* burdens *in vivo* (Fig. 3B). In the ST784 PBS group (day 6 versus day 0), the relative abundances of four strains (Clostridium clostridioforme, Muribaculum intestinale, Lactobacillus reuteri, and Enterococcus faecalis) were altered by the presence of *Salmonella* (Tables S3). Interestingly, in ST784 FOP samples, the abundances of C. clostridioforme and M. intestinale were not significantly altered, while the abundances of L. reuteri and E. faecalis were. These results indicate that the latter two strains were strongly associated with the burden of *Salmonella*, while the other two were not, suggesting that FOP prevented their variation (Table S3). These data are consistent with those previously obtained with an *in vitro* human model system (24) showing that FOP administration does not alter the resident gastrointestinal microbial community.

### Decreasing the FOP dose does not reduce its *in vivo* efficacy.

**FIG 4:**
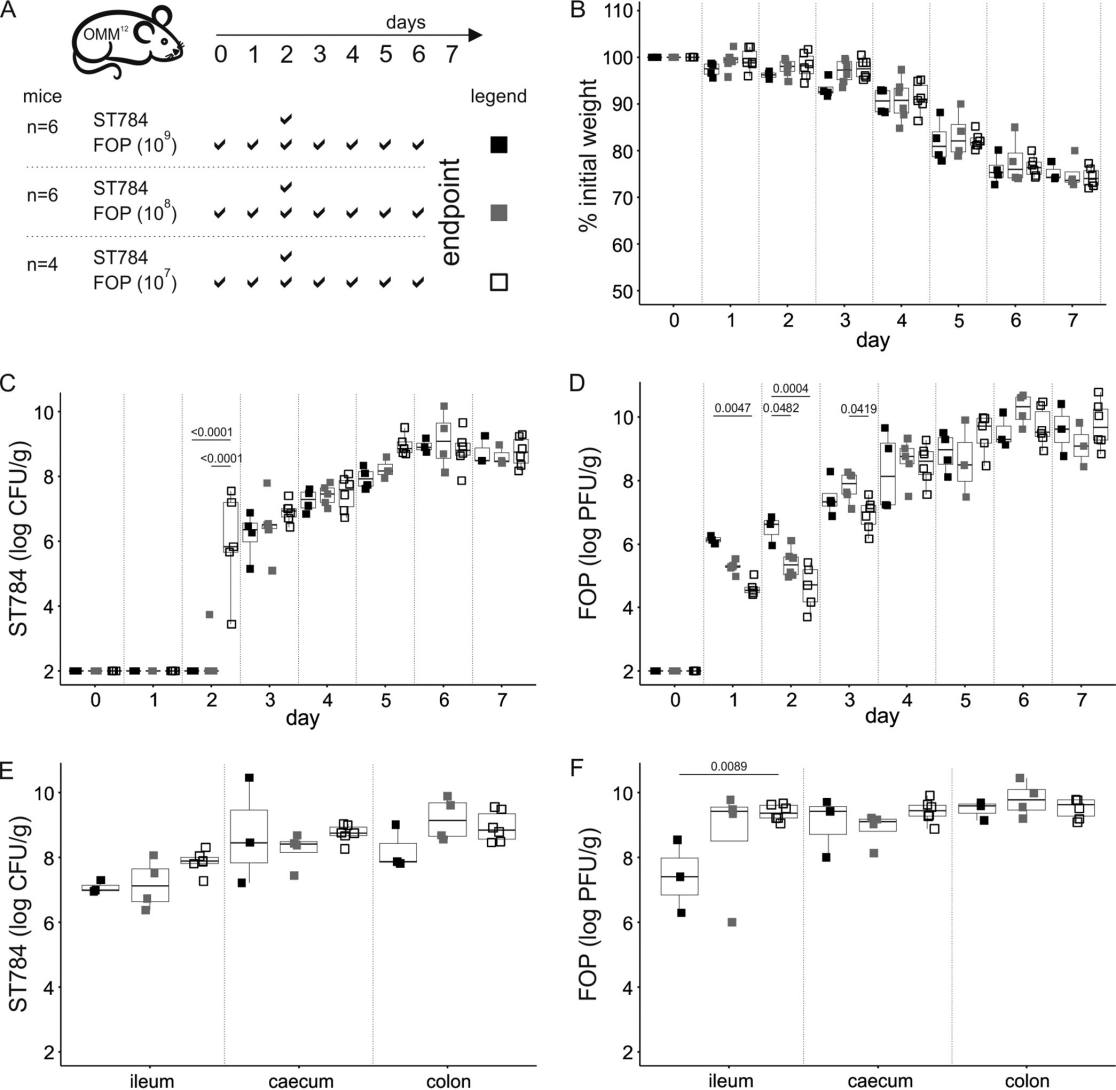
Dose dependence of effect of FOP on ST784 burden in OMM^12^ mice. (A) Experimental design. OMM^12^ mice (*n* = 4 to 6) were orally administered the indicated doses of FOP (10^7^ PFU, white squares; 10^8^ PFU, gray squares; 10^9^ PFU, black squares) on the indicated days. On day 2, all mice were challenged with strain ST784 (1 × 10^8^ CFU). (B) Mice were weighed daily. Shown are the percentages of weight loss compared to starting weights of OMM^12^ mice over time. (C and D) The amounts of ST784 (CFU) (C) and phages (PFU) (D) in OMM^12^ mouse feces were quantitated daily (day 2 corresponds to 3 h after ST784 challenge). (E and F) Intestinal organs were collected on day 6, and the amounts of ST784 (CFU) (E) and phages (PFU) quantitated. Statistical analyses are described in Materials and Methods and reported in Table S4.

To assess whether there was a dose-dependent effect on the ability of FOP to reduce *Salmonella in vivo*, three groups of 4 to 6 OMM^12^ mice were orally administered FOP twice a day for 7 days at different doses with 10-fold reductions between doses, ranging from 1 × 10^9^ to 1 × 10^7^ PFU (Fig. 4A). All animals received a single dose of ST784 (1 × 10^8^ CFU) on day 2. No differences in mouse behavior or weight were observed between the three groups (Fig. 4B and Table S4). At 3 h post-*Salmonella* administration, all mice that received the lowest dose of FOP (1 × 10^7^ PFU) had detectable levels of *Salmonella* in feces, while only one mouse in the group receiving the middle dose (1 × 10^8^ PFU) and none receiving the highest dose (1 × 10^9^ PFU) had detectable ST784 counts (Fig. 4C and Table S4). This indicates that the highest prophylactic dose of FOP had a stronger impact on the ST784 inoculum. During the next 3 days (days 3 to 5), fecal levels of *Salmonella* rose in all three groups with no significant differences; however, the levels trended inversely proportional to the FOP dose (Fig. 4C and Table S4). During the last 2 days (days 5 and 6), *Salmonella* levels reached a median value of 10^8^ CFU/g in feces and the difference between the groups vanished. Fecal levels of phages showed initial differences between the three groups on days 1 and 2 in agreement with the administered doses. Then, over the next several days, phage levels progressively increased, mirroring *Salmonella* fecal levels, with diminishing differences between the dosing groups until reaching a plateau where the median phage level was approximately 10^9^ PFU/g (Fig. 4D and Table S4). We measured *Salmonella* (Fig. 4E) and phage (Fig. 4F) levels from gut sections on day 7 (1 day later than the experiments whose results are shown in Fig. 1 and 2). No significant differences in levels of ST784 between the three gut compartments were discerned, as expected from fecal contents (Fig. 4C), while a significant difference in phage counts could only be detected in the ileum between the highest and lowest dose groups (Fig. 4F and Table S4).

## DISCUSSION

Several phage products have been granted generally recognized as safe (GRAS) status by the U.S. Food and Drug Administration (FDA), which is in agreement with a large body of literature that has shown that phages are innocuous when ingested by humans (27–29). This is not surprising, given the large and diverse amount of phages already present in the human gut (10^15^) (30). Nevertheless, the characterization of any new phage product for human applications must include information on safety as well as efficacy. Here, we evaluated the prophylactic application of FOP, a novel phage cocktail designed to target FBDs, including gut infections of *Salmonella*, which remains one of the most common causes of FBD worldwide. We attempted to mimic a real-life situation where individuals at risk, residing in the close proximity of an ongoing foodborne epidemic, would prophylactically take a phage product to lower the probability of contracting a disease. For this purpose, we used OMM^12^ mice that do not provide colonization resistance to *Salmonella* and therefore do not require antibiotic treatment (22, 23), in order to perform our study with an *in vivo* controlled-microbiota environment.

Given the expected rate of passive transit of phages in the guts of mice (31), we administered phages twice a day to maintain a high level of phages in order to maximize their impact on incoming *Salmonella* bacteria. The safety of such repetitive treatment was confirmed by the lack of any abnormal behavior or clinical symptoms, as well as the quantitation of an intestinal inflammatory marker (lipocalin-2), which remained below the threshold of inflammation. In addition, no impact on the microbiota structure was observed following 48 h of such treatment. Altogether, these data confirm that FOP is equally as safe as any of the three GRAS phage cocktails that compose it (i.e., EcoShield, ListShield, and SalmoFresh) (32).

We show that the prophylactic administration of SalmoFresh or FOP delayed the onset and lessened the most severe symptoms of *Salmonella* infection compared to the time of onset and severity of symptoms in PBS controls. Remarkably, the nearly 2-log decrease in CFU in the feces of mice that received FOP was also observed in each gut section, showing that phages can kill *Salmonella* with the same efficacy throughout the intestine. When comparing three different doses of FOP, we observed that 3 h after the ST784 challenge, the highest dose was sufficient to reduce the shedding of the inoculum to below the limit of detection. This rapid efficacy is congruent with data obtained with an *in vitro* human gut simulation model, which showed that FOP treatment killed ≥90% of ST784 after 5 h while simultaneously inhibiting the ability of ST784 to invade human intestinal cells (24). This strong impact on the *Salmonella* inoculum is encouraging for the prophylactic application of phages. Indeed, the *Salmonella* dose used in mice (1 × 10^8^ CFU for a 20-g mouse) would correspond to a dose of 3.5 × 10^11^ CFU for a 70-kg human, which is several log higher than the infective dose for nontyphoidal salmonellosis (<10^3^ CFU) (33, 34), as well as the most probable numbers (MPNs) of contaminating bacteria in retail settings, which are estimated to be even lower (35–37). Therefore, an FOP dose of 1 × 10^9^ PFU would still remain several log higher than the target dose of *Salmonella*.

During all experiments, and more strikingly during the FOP dose experiments, the fecal levels of phages rose rapidly following ST784 challenge, demonstrating that orally administered FOP is actively replicating in the gut at the expense of ST784. Interestingly, during the dose experiment, within 24 h there was already no difference between the phage levels in mice that received the middle and the high doses, and within another 24 h, the phage levels resulting from the three doses were undistinguishable, while all three doses were significantly different before the challenge. These data showed that within 48 h, a 100-fold-lower dose of FOP reached a similar density in the gut that was associated with an equivalent impact on the *Salmonella* burden.

The relative abundances of 4 of the 12 intestinal bacteria of OMM^12^ mice were altered by increased levels of ST784 *Salmonella*. However, the FOP treatment limited these variations to increased abundances of L. reuteri and E. faecalis, suggesting that these two strains benefit from the presence of *Salmonella*. These observations illustrate that the growth of a pathogen has a direct impact on microbiota structure and that a phage intervention can limit such impact. They also demonstrate that murine gnotobiotic models, despite their limitations, are suitable to study mechanisms involved in the role of the gut microbiota during infection by *Salmonella* (22) and, potentially, other intestinal pathogens.

Finally, we showed that the prophylactic administration of phage products like FOP lowers the burden of a foodborne intestinal pathogen and could represent a strategy to reduce the dissemination of FBD during an outbreak. Lowering the spread of the contamination until the identification of its source, which can last several weeks, would have direct economic impact (38). In addition, the use of phages instead of antibiotics will be beneficial to the resident microbiota and will not increase the selection for antibiotic-resistant pathogens.

## MATERIALS AND METHODS

### Ethics statement.

All animal experiments were approved by the committee on animal experimentation at the Institut Pasteur (Paris, France) and by the French Ministry of Research. A total of 68 OMM^12^ (C57BL/6J) mice from 7 to 9 weeks old, bred at Institut Pasteur, were used.

### Bacterial strains and phage products.

S. enterica subsp. *enterica* serovar Typhimurium strain ST784 was obtained from Intralytix, Inc. (Columbia, MD), and routinely cultured in lysogeny broth (LB), on LB agar, or on Drigalski agar (Bio-Rad, Hercules, CA) at 37°C. For oral administration of ST784, an overnight liquid culture in LB was diluted 1/10 in sucrose bicarbonate buffer (20% sucrose and 2.6% sodium bicarbonate, pH 8). SalmoFresh (25, 39) and FOP (24) phage preparations were obtained from Intralytix. SalmoFresh is a commercially available phage cocktail consisting of 6 lytic phages at approximately equivalent titers targeting *Salmonella* spp., 4 of which infect strain ST784 with similar efficiencies. The FOP phage cocktail, which includes 15 phages at approximately equivalent titers, was prepared as described previously (24). It is a combination of three previously described commercial FDA-affirmed generally recognized as safe (GRAS) phage cocktails (i.e., ListShield, EcoShield PX, and SalmoFresh) that, combined, target Listeria monocytogenes, E. coli (STEC), and *Salmonella*, respectively (24). Before use, the phage cocktails were diluted in PBS to the indicated concentrations, which were calculated from the titration of these products on strain ST784. *Salmonella*-specific-phage quantification was performed by spotting serial dilutions of fecal or intestinal-section samples on LB plates overlaid with strain ST784 (24).

### Murine model.

In all experiments, mice were randomly assigned to a group, and each group included approximately the same numbers of male and female animals kept in separate cages. Every day, mice were observed and weighed and feces were collected. Fecal pellets were transferred into preweighed, sterile, 2 ml tubes, weighed, and then resuspended in 1 ml of PBS. Serial dilutions in PBS were performed and plated onto Drigalski plates for *Salmonella* quantitation (CFU) and onto LB plates overlaid with ST784 for phage titration (PFU).

SalmoFresh, FOP, or PBS was administered by oral gavage in 200 μl twice daily, 6 h apart. The dose of phages administered through each gavage was 1 × 10^9^ PFU, or lower as specified. Strain ST784 (1 × 10^8^ CFU) was administered by oral gavage in 200 μl on day 2, 3 h after the first phage gavage on that day. Control mice not receiving ST784 received PBS instead. At the end of all experiments, mice were sacrificed by cervical dislocation, and intestinal sections (i.e., ileum, cecum, and colon) were collected without the separation of luminal and mucosal contents. These sections were homogenized in PBS using the gentleMACS OctoDissociator (Miltenyi Biotec), serially diluted in PBS, and plated on both Drigalski plates and LB plates overlaid with strain ST784.

### Lipocalin-2 assay.

Frozen supernatants of fecal samples resuspended in PBS were thawed before lipocalin-2 quantification using a commercial enzyme-linked immunosorbent assay (ELISA) kit (catalog number DY1857; R&D Systems) according to the manufacturer’s instructions. The threshold of inflammation (5 ng/g of feces) was defined according to the method in reference 26.

### 16S rRNA gene analysis.

Resuspended fecal samples (500 μl) were centrifuged at 8,000 × *g* for 10 min, and the supernatant removed. Pellets were resuspended in 500 μl of lysis buffer (500 mM NaCl, 50 mM Tris-HCl, pH 8.0, 50 mM EDTA, 4% sodium dodecyl sulfate) and incubated for 15 min at 50°C (40). Then, 100 μl of lysozyme (25 mg/ml) was added and samples were incubated at 37°C for 2 h. DNA extraction was performed using the Maxwell 16 tissue DNA purification kit (Promega). Amplicon libraries targeting the V3-V4 16S rRNA gene region were then constructed using Illumina primers (forward primer, 5′-TCGTCGGCAGCGTCAGATGTGTATAAGAGACAGCCTACGGGNGGCWGCAG-3′, and reverse primer, 5′-GTCTCGTGGGCTCGGAGATGTGTATAAGAGACAGGACTACHVGGGTATCTAATCC-3′) and amplified by PCR for 25 cycles. The libraries were sequenced on an Illumina MiSeq instrument (2 × 300 bp). FastQC (http://www.bioinformatics.babraham.ac.uk/projects/fastqc/) was used for the quality control of the reads. Read filtering, operational taxonomic unit (OTU) clustering, and annotation were performed with the MASQUE pipeline (https://github.com/aghozlane/masque). All statistical analyses were performed with SHAMAN (http://shaman.c3bi.pasteur.fr) as previously described (41).

### Statistical analysis.

Statistical analysis on the numbers of bacteria (CFU) and phages (PFU), as well as mouse weights, were carried out using the lme4 and lmerTest packages of R (42, 43). Both CFU and PFU were log_10_ transformed prior to analysis. Given the nonlinearity of responses, the day at which a measure was performed was considered a categorical variable. Linear mixed models were used to account for random experimental effects (i.e., individuals, experiments, and cage effects). Overall effects were assessed with analysis of variance (ANOVA) and *post hoc* Tukey’s comparisons and were performed using the lsmeans R package (44). A *P* value of <0.05 was considered statistically significant.
