# Supplementary material for: Prophylactic Administration of a Bacteriophage Cocktail Is Safe and Effective in Reducing Salmonella enterica Serovar Typhimurium Burden in Vivo
Source: Microbiol Spectr. 2021 Aug 25;9(1):10.1128/spectrum.00497-21. doi: 10.1128/spectrum.00497-21 (PMC8552648; doi:10.1128/spectrum.00497-21)
Supplement: SUPPLEMENTAL FILE 5 — Supplemental material. Download SPECTRUM00497-21_Supp_5_seq12.pdf, PDF file, 0.1 MB [file spectrum00497-21_supp_5_seq12.pdf]

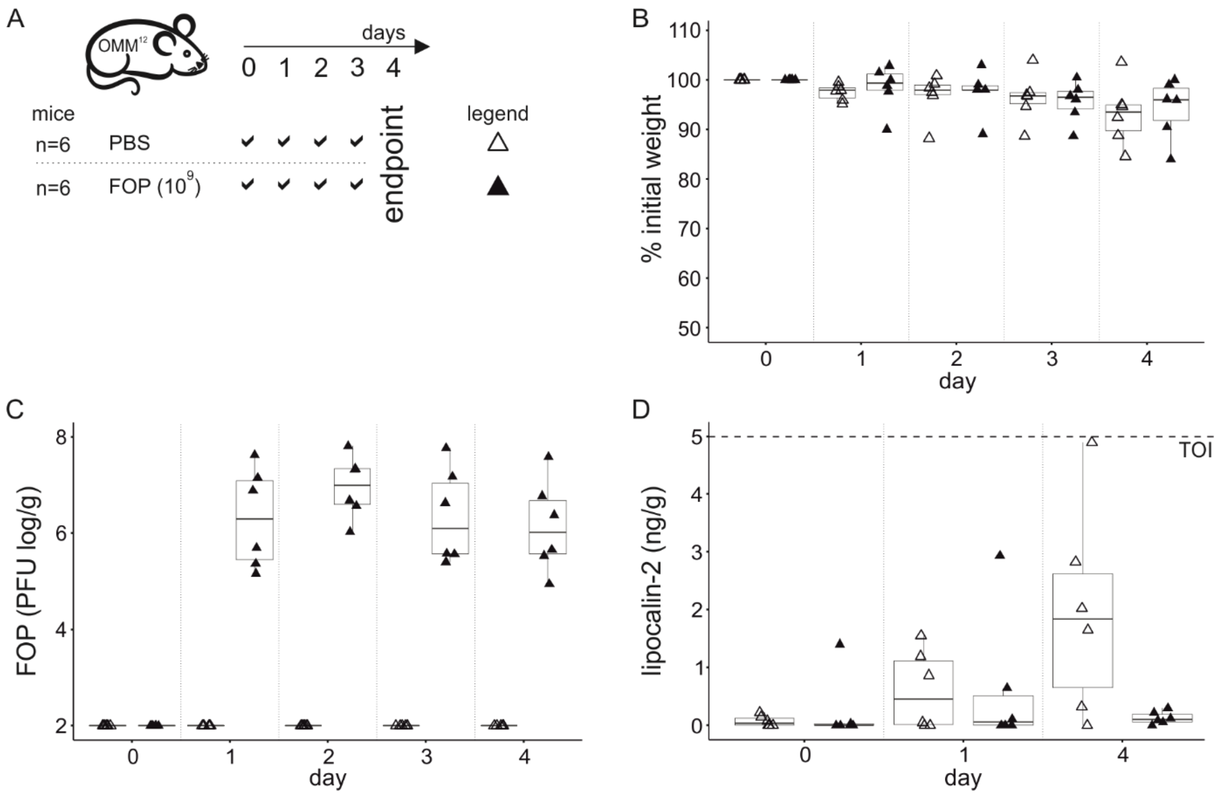

Figure S1. Repeated administration of FOP to OMM^{12} mice is safe

A. Experimental design. OMM^{12} mice (n = 6) were orally administered FOP (10^9 PFU), black triangles) or PBS (white triangles) on the indicated days. **B.** Mice were weighed daily. Shown is the percentage of weight loss compared to starting weight of OMM^{12} mice over time. **C.** The amount of phage in OMM^{12} mice feces were quantitated daily. Shown are the PFU counts in OMM^{12} mice that received the indicated doses of FOP. **D.** Fecal lipocalin-2 was quantitated (ng/g) over time. The horizontal dash line represents the threshold of inflammation (TOI) as defined in (26)**.**
